# Transcriptional coactivator CBP upregulates hTERT expression and tumor growth and predicts poor prognosis in human lung cancers

**DOI:** 10.18632/oncotarget.2430

**Published:** 2014-09-03

**Authors:** Wei Guo, Jianjun Lu, Meng Dai, Taihua Wu, Zhenlong Yu, Jingshu Wang, Wangbing Chen, Dingbo Shi, Wendan Yu, Yao Xiao, Canhui Yi, Zhipeng Tang, Tingting Xu, Xiangsheng Xiao, Yuhui Yuan, Quentin Liu, Guangwei Du, Wuguo Deng

**Affiliations:** ^1^ Institute of Cancer Stem Cell & First Affiliated Hospital, Dalian Medical University, Dalian, China; ^2^ Sun Yat-sen University Cancer Center, State Key Laboratory of Oncology in South China; Colaborative Innovation Center of Cancer Medicine, Guangzhou, China; ^3^ Department of Thoracic Surgery, The First Affiliated Hospital, Sun Yat-sen University, Guangzhou, China; ^4^ Department of Integrative Biology and Pharmacology, The University of Texas Health Science Center, Houston, Texas, USA

**Keywords:** CBP, hTERT, promoter, Sp1, lung cancer

## Abstract

Upregulated expression and activation of human telomerase reverse transcriptase (hTERT) is a hallmarker of lung tumorigenesis. However, the mechanism underlying the aberrant hTERT activity in lung cancer cells remains poorly understood. In this study, we found the transcriptional co-activator CBP as a new hTERT promoter-binding protein that regulated hTERT expression and tumor growth in lung adenocarcinoma cells using a biotin-streptavidin-bead pulldown technique. Chromatin immunoprecipitation assay verified the immortalized cell and tumor cell-specific binding of CBP on hTERT promoter. Overexpression of exogenous CBP upregulated the expression of the hTERT promoter-driven luciferase and endogenous hTERT protein in lung cancer cells. Conversely, inhibition of CBP by CBP-specific siRNA or its chemical inhibitor repressed the expression of hTERT promoter-driven luciferase and endogenous hTERT protein as well as telomerase activity. Moreover, inhibition of CBP expression or activity also significantly reduced the proliferation of lung cancer cells *in vitro* and tumor growth in an xenograft mouse model *in vivo*. Immunohistochemical analysis of tissue microarrays of lung cancers revealed a positive correlation between CBP and hTERT. Importantly, the patients with high CBP and hTERT expression had a significantly shorter overall survival. Furthermore, CBP was found to interact with and acetylate transactivator Sp1 in lung cancer cells. Inhibition of CBP by CBP-specific siRNA or its chemical inhibitor significantly inhibited Sp1 acetylation and its binding to the hTERT promoter. Collectively, our results indicate that CBP contributes to the upregulation of hTERT expression and tumor growth, and overexpression of CBP predicts poor prognosis in human lung cancers.

## INTRODUCTION

Telomeres and telomerase have been found to play important roles in tumorigenesis and development [1,2,3]. Telomeres are repetitive nucleotide sequences located at the ends of linear chromosomes in eukaryotic cells, and are required for maintaining integrity of the chromosomes. They are shortened at each cell division. Telomerase is a ribonucleoprotein complex composed of RNA and human telomerase reverse transcriptase (hTERT). It uses its own RNA as template to synthesize telomeres, and then add telomeres to the ends of chromosomes to extend the shortened telomeres and enhance cell proliferation, thereby promoting cellular immortalization. High activity of telomerase has been implicated in maintaining telomere stability, genome integrity, cell activity, potential long-term proliferative ability and other aspects in cancer cells [4,5]. Telomerase activity is usually low or under detection in normal human cells except human embryonic stem cells and germ cells, but it is highly activated in the immortalized cells and more than 85% of tumor cells during malignant transformation [6-12].

As the catalytic subunit of telomerase, hTERT determines the activity of telomerase[13]. Multiple transcription factor binding sites exist in the hTERT promoter region, and it is recognized that its expression is tightly controlled by these regulatory factors [14]. Many oncogenes, such as c-Myc, Sp1, HIF-1, AP-2, Estrogen receptor, and IRF1, could function as transcriptional factors to stimulate the expression of hTERT, while many tumor suppressors, such as p53, WT1, Menin and SMAD3, inhibit hTERT transcriptional activation by binding to special promoter sites [15-17]. In addition, the demethylation of histones at promoter region could also promote the expression of hTERT under the action of histone acetyltransferase (HAT) [18]. However, how hTERT activity is silenced in normal cells and reactivated during the process of carcinogenesis is still unclear. We speculate that certain transcription factors or regulatory factors differentially or selectively bind to the hTERT promoter region in human cancer cells compared with normal cells to promote hTERT expression and telomerase activity, and thus to regulate tumor development.

CREB-binding protein (CBP) is a highly conserved transcriptional coactivator, which shares similar structure with its paralog, E1A-binding protein (p300). They both have four separate transactivation domains that interact with general transcription factors as well as DNA-binding transcription activators to mediate the recruitment of basal transcriptional machinery to the promoter and increase the expression of their target genes [19,20]. CBP/p300 also contains histone acetyltransferase (HAT) activity, which is able to relax chromatin's superstructure and promote expression of the proximal genes. In addition, the HAT activity of CBP/p300 could also acetylate some transcription factors and further modulate their functions [21]. Based on their interactions with histones and a large number of transcriptional factors, CBP/p300 have been found to be implicated in many complex pathological and physiological processes, including cancers [22, 23]. By activating some oncogenes, such as c-Myc, c-Myb and AR, CBP/p300 can promote cell proliferation and cancer development. For example, CBP/p300 promotes prostate cancer progression by activating AR-dependent transcription [24]. CBP is also associated with breast cancer and acute lymphoblastic leukemia (ALL) drug resistance by interacting with β-catenin [25,26]. However, whether CBP or p300 regulates lung tumorigenesis and development by modulating hTERT signaling as well as the exact underlying mechanisms are largely unknown.

In this study, we have found the high expression of CBP and hTERT in lung tumor cells and tissues. Therefore, we hypothesize that the differentially expressed CBP may specifically bind to the hTERT promoter to regulate hTERT expression and tumor growth in lung cancer cells. Previous study has also shown that hTERT expression is dependently regulated by activating specificity protein 1 (Sp1) in human lung cancer cells [27]. Given these findings and the possibly potential correlation between CBP and hTERT expression in lung cancer, in this study, we investigated whether CBP might play a regulatory role in hTERT expression in lung cancer cells as transcriptional co-activator through cooperation with some specific transcriptional factors such as Sp1. Since hTERT expression is closely related to carcinogenesis and strictly controlled at the transcription level, our findings will reveal the new function of CBP as a tumor-specific transcriptional co-activator to regulate hTERT expression and tumor growth in human lung cancer cells.

## RESULTS

### Detection of CBP as a hTERT promoter-binding protein in lung cancer cells

**Figure 1:**
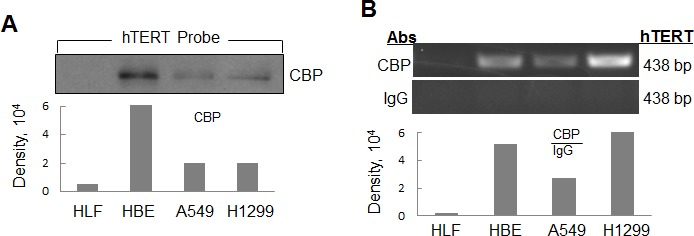
Identification of CBP as a hTERT promoter-binding protein in lung cancer cells (A) Streptavidin-agarose bead pulldown assay with hTERT promoter (-378 to +60) as probes was done in human normal lung cells and lung cancer cells. The pulled down proteins were tested by immunoblot using antibodies against CBP. (B) Chromatin immunoprecipitation assay was done with normal lung cells and lung adenocarcinoma cells using antibodies against CBP. PCR products were separated on 1% agarose gels. The last lane represents the IgG control. The displayed gels were representative of 2-3 independent experiments. Densitometric analysis was used to analyze quantitatively the binding activity of CBP protein on hTERT promoter.

We designed and synthesized a 438 bp biotin-conjugated double stranded DNA oligonucleotide probe corresponding to −378 to +60 site of hTERT gene promoter region. The biotinylated hTERT promoter probe can be specifically connected to the agarose beads coated with streptavidin. We then incubated the cell nuclear extract proteins with streptavidin-agarose beads coated with the hTERT probe. The hTERT promoter-binding proteins were purified and separated by SDS-PAGE. The proteins of interests were detected by immunoblot analysis using the specific antibodies. CBP was identified as a hTERT promoter-binding protein and detected in the complexes prepared from human lung cancer H1299 and A549 cells and the immortalized HBE cells, but not from the normal lung HLF cells (Fig. 1A).

To further verify that CBP binds to the hTERT promoter specifically in cancer and immortalized cells, we also performed chromatin immunoprecipitation (ChIP) assay to detect the binding of CBP to the endogenous hTERT promoter. As we expected, more chromatin hTERT promoter DNA were amplified in lung cancer A549 and H1299 cells and immortalized HBE cells than those in normal lung HLF cells when a CBP antibody was used in the ChIP assay (Fig. 1B), whereas nearly no DNA-binding was detected when a control normal IgG was used. The results demonstrated the tumor and immortalized cell-specific binding of CBP to the hTERT promoter in lung cancer cells.

### Regulation of hTERT promoter activity by CBP in lung cancer cells

**Figure 2:**
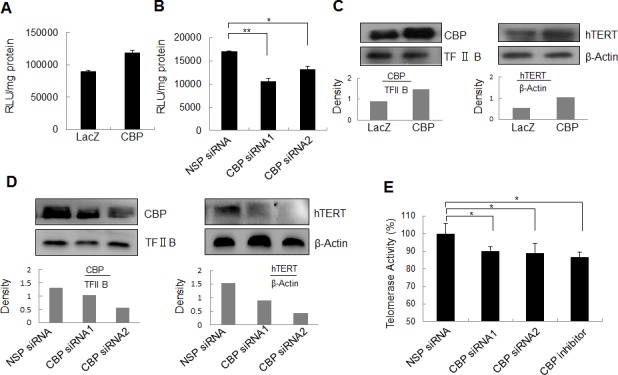
The effect of CBP on hTERT promoter activity, hTERT protein expression and telomerase activity (A) Lung cancer cells were co-transfected with the plasmids of hTERT promoter driven-luciferase and pCDNA3.1-CBP or pCDNA3.1-Lac Z for 48 h followed by a dual-luciferase assay. The relative luciferase intensity per mg protein was calculated in the treated cells. (B) Lung cancer cells were co-transfected with hTERT promoter driven-luciferase and CBP siRNA or control siRNA for 48 h followed by a dual-luciferase assay. The relative luciferase intensity per mg protein was calculated in the treated cells. (C) Up-regulation of hTERT protein expression by the overexpression of CBP. H1299 cells were treated with pcDNA3.1-CBP for 48 h, and the expression of CBP itself in the nucleus and hTERT protein in the cytoplasm were analyzed by Western blot in lung cancer cells. (D, E) Downregulation of hTERT protein expression by the knockdown of CBP expression. H1299 cells were transfected with a CBP-specific siRNA for 48 h, the expression of CBP itself in the nucleus and hTERT protein in the cytoplasm were analyzed by Western blot (D), and the telomerase activity was assessed (E). All of the measurements represent the means ± SE of three independent experiments. *, P < 0.05, significant differences between treatment groups and DMSO control groups.

To understand whether CBP functions as a transcriptional co-activator to regulate hTERT promoter activity, lung cancer cells H1299 were transfected with plasmids expressing CBP and a luciferase reporter driven by the hTERT promoter, and then assayed for the activity of luciferase reporter. As shown in Fig. 2A, overexpression of CBP upregulated the activity of hTERT promoter-driven luciferase in H1299 cells co-transfected with CBP and hTERT-luciferase plasmids compared with that in cells co-transfected with LacZ and hTERT-luciferase plasmids. In contrast, siRNA knockdown of CBP expression or CBP inhibitor treatment significantly attenuated the activity of hTERT promoter-driven luciferase in H1299 cells co-treated with hTERT-luciferase and a CBP-specific siRNA or inhibitor compared with those in cells co-treated with the non-specific siRNA (NSP siRNA) and hTERT-luciferase plasmid (Fig. 2B). These results demonstrated that CBP might serve as a transcriptional co-activator in the activation of hTERT promoter in lung cancer cells.

### Regulation of hTERT expression and telomerase activity by CBP in lung cancer cells

To further confirm the role of CBP in regulating hTERT expression, we evaluated the effect of CBP on hTERT protein expression and telomerase activity in lung cancer H1299 cells. Ectopic expression of CBP increased not only CBP itself, but also hTERT protein levels in H1299 cells (Fig. 2C). Conversely, the downregulation of CBP expression by CBP siRNA suppressed hTERT protein expression (Fig. 2D). Moreover, knockdown of CBP by siRNA or inhibition of its activity by a CBP-selective inhibitor significantly repressed the telomerase activity in H1299 cells (Fig. 2E). These findings suggest that CBP exerts a positive role in the regulation of hTERT protein expression and telomerase activity.

### Regulation of lung cancer growth by CBP *in vitro* and *in vivo*

**Figure 3:**
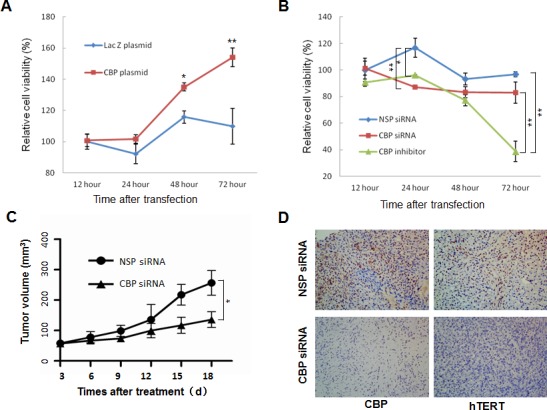
The effect of CBP on lung tumor growth *in vitro* and *in vivo* (**A**) H1299 cells were transfected with CBP overexpression vector pcDNA3.1-CBP. At different time points after transfection, cell viability was measured by MTT assay. (**B**) H1299 cells were treated with CBP specific siRNA or inhibitor. At different time points after treatment, cell viability was measured by MTT assay. The mean and SE obtained from three independent experiments are plotted (*, *P* < 0.05,**, *P* < 0.01). (C) The nude mice containing xenografts of human lung cancer were intratumorally treated with non-specific control siRNA or CBP-specific siRNA, and the tumor volumes ± SE were calculated at different days after treatment. (N=5; *, P < 0.05). (D) Immunohistochemistry of CBP and hTERT from tumor xenografts in nonspecific control siRNA- and CBP-specific siRNA-treated nude mice (400× magnification).

Telomerase is implicated in tumor progression and has become a potential target of cancer therapy. Our data so far have indicated that CBP might be involved in lung cancer cell proliferation through the regulation of hTERT. To test this, we first tested the effect of the CBP on cell proliferation in H1299 cells *in vitro*. Overexpression of CBP effectively promoted H1299 cell proliferation as compared to the control LacZ group(Fig. 3A). By contrast, inhibition of CBP expression by a CBP-specific siRNA or a CBP-selective inhibitor significantly inhibited the proliferation of H1299 cells (Fig. 3B). These results suggest an important role of CBP in sustaining lung cancer cell proliferation.

We then evaluated the effect of CBP on lung tumor growth using an xenograft mouse model bearing lung tumors. As shown in Fig. 3C, knockdown of CBP expression dramatically suppressed tumor growth in human lung cancer mouse model *in vivo* in comparison with the non-specific control siRNA (NSP-siRNA) treatment, demonstrating the role of CBP in the regulation of lung cancer growth *in vivo*.

We also analyzed the correlation between CBP and hTERT protein expression in tumor tissues of xenografts by immunohistochemical staining analysis. The results showed that downregulation of CBP by siRNA markedly decreased hTERT protein expression compared with the control NSP siRNA treatment in xenografts (Fig. 3D), suggesting that CBP-mediated tumor growth *in vivo* is likely regulated by hTERT.

### Overexpression of CBP and hTERT in lung cancer cells

**Figure 4:**
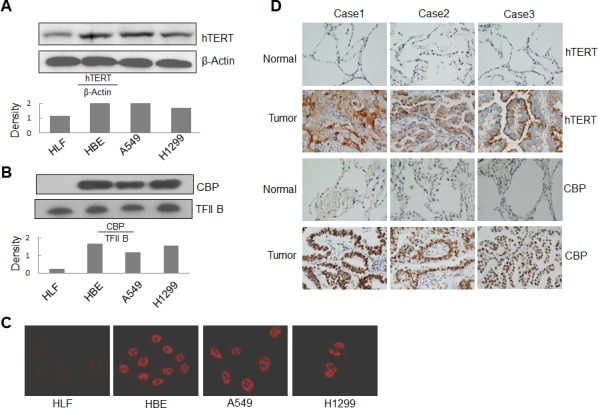
Overexpression of CBP and hTERT in lung cancer cells and tumor tissues (A) Western blot analysis of hTERT expression from cytoplasmic lysate in human lung normal and cancer cells. β-Actin was used as control. (B) Western blot analysis of CBP expression from nuclear lysate in human lung normal and cancer cells. TFIIB was used as control. (C) The expression and distribution of CBP in human lung normal and cancer cells through immunofluorescent analysis. (D) The expression of hTERT and CBP protein in tumor tissues of patients with lung adenocarcinomas and corresponding adjacent normal lung tissues by immunohistochemistry analysis (magnification, ×200).

To determine how tumor cells differentially activate hTERT expression, we detected the expression of hTERT and transcriptional co-activator CBP at protein levels in human lung cancer and normal cells by Western blot analysis. The hTERT expression was detected in cytosol (Fig. 4A), and CBP was detected in nuclei (Fig. 4B). Compared to the normal HLF cells, CBP protein was obviously highly expressed in lung cancer cell lines A549 and H1299 and in the immortalized cell line HBE (Fig. 4B). Similarly, hTERT protein was also expressed at a comparatively high level in A549, H1299 and HBE cells (Fig. 4A).

We also tested the expression and localization of CBP by an immunofluorescent staining. Consistent with the results from Western blot analysis, nearly no expression of CBP was found in normal HLF cells, but high expression was found in the nuclei of both A549 and H1299 cells and in the immortalized cell line HBE (Fig. 4C).

To further investigate the regulation of hTERT by CBP in lung cancer, we next analyzed the expression of CBP and hTERT proteins in lung tumor and normal lung tissues of patients by immunohistochemical staining. As shown in Fig. 4D, both CBP and hTERT were highly expressed in lung tumor tissues compared with the normal lung tissues in all three tested patients. These results indicate the very possible positive correlation between CBP and hTERT, and verified the potential regulation of hTERT expression by CBP in lung cancer.

### Positive correlation between CBP and hTERT expression in lung cancers and their association with prognosis of the patients with lung adenocarcinomas

**Figure 5:**
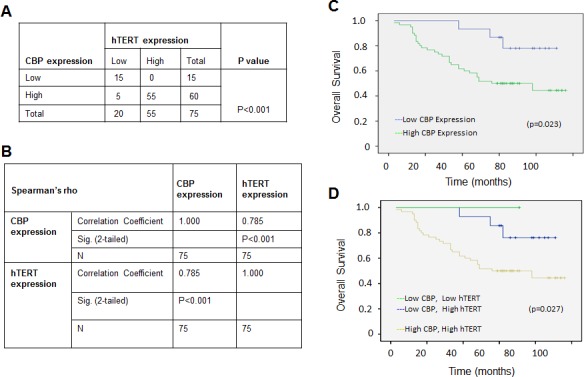
The positive correlation between CBP and hTERT protein expression in lung adenocarcinoma specimens, and a comparatively poor prognosis indicated by the higher expression of CBP and hTERT (A) The correlation between CBP and hTERT protein in lung adenocarcinoma tissues (P < 0.001, χ2 tests). (B) The correlation between CBP and hTERT protein in lung cancer tissues. (P<0.01, 2-tailed test). (C) Kaplan–Meier analysis of overall survival of lung cancer patients with different CBP expression (p<0.05, log-rank test). (D) Kaplan–Meier analysis of overall survival of lung cancer patients with different CBP and hTERT expression (p<0.05, log-rank test).

We analyzed the expression of CBP and hTERT proteins in lung tumor and normal lung tissues by immunohistochemical assay in 75 cases of patients with lung cancers. The percentage of the cases with both CBP and hTERT high expression nearly reached 73% based on the total number of the studied cases (Fig. 5A). Furthermore, the analysis by a Pearson's correlation coefficient test showed that CBP expression was statistically positively correlated with hTERT expression, giving a Pearson R value of 0.785 (Fig. 5B), suggesting a high significance of CBP expression level and its positive correlation with hTERT in lung adenocarcinoma development.

We further analyzed the effect of CBP expression on the survival rate of patients with lung adenocarcinomas. The overall survival curve analysis indicated a relative poor prognosis in lung adenocarcinomas patients with high CBP expression compared to those with weak CBP expression (Fig. 5C). Moreover, lung adenocarcinomas patients with very weak or negative CBP and hTERT expression have nearly 100% survival rate of 8 years after diagnosis, but the survival rate for the patients with both high CBP and hTERT expression was lowered to 50% 5 years after diagnosis (Fig. 5D), suggesting the important role of CBP expression and its association with hTERT in predicting the prognosis of patients with lung adenocarcinomas.

### Interaction of CBP with Sp1 and AP-2 in lung cancer cells

**Figure 6:**
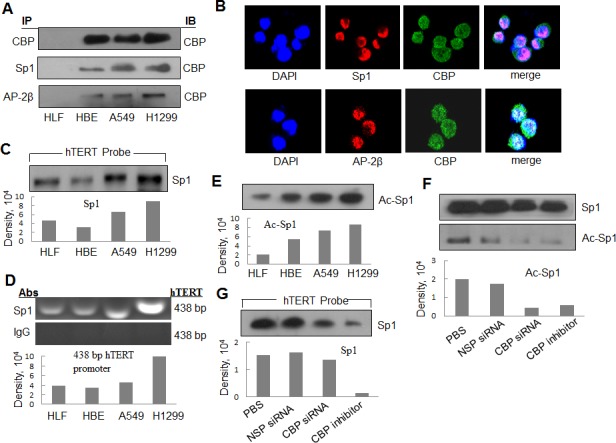
The interaction of CBP with Sp1 and AP-2 and the acetylationt of Sp1 by CBP in lung cancer cells (A) The nuclear extracts of human lung normal and cancer cells were prepared for immunoprecipitation using an antibody against Sp1 or AP-2β and then evaluated by immunoblot using antibody against CBP. (B) Human lung cancer H1299 cells grown on chamber slides were cultivated for 24 h, and the subcellular localization and the colocalization of CBP with Sp1 or AP-2β were examined by confocal microscopy analysis with a confocal microscope. More than 100 cells were inspected per experiment, and cells with typical morphology were presented. (C) Streptavidin-agarose bead pulldown assay with hTERT promoter (-378 to +60) as probes was done. Sp1 was tested in the pulled down proteins by immunoblot using antibody against Sp1. (D) Chromatin immunoprecipitation assays were done using antibody against Sp1. PCR products of hTERT promoter (-378 to +60) were separated on 1% agarose gels. The last lane represents the IgG control. (E) Immunoprecipitation was performed using antibody against Sp1. The acetylated Sp1 was determined by immunoblot using the antibody against acetylation. (F) Immunoprecipitation was performed in human lung cancer cells (H1299) treated by non-specific siRNA or CBP specific siRNA or inhibitor using antibody against Sp1. The acetylated Sp1 was tested by immunoblot using antibody against acetylation. (G) Streptavidin-agarose bead pulldown assay with hTERT promoter (-378 to +60) as probes was done in lung cancer cells (H1299) treated by non-specific siRNA or CBP specific siRNA or CBP-specific inhibitor. The level of Sp1 in the pulled down proteins was determined by immunoblot. Densitometric analysis was used to analyze quantitatively the binding activity and acetylation level of Sp1 proteins.

CBP is a transcriptional co-activator that binds to the transactivator-promoter complex and regulates the expression of its target genes. To determine whether CBP specifically binds to hTERT promoter through serving as a transcriptional co-activator and interacting with the transactivators which bind to the hTERT promoter and differentially activates hTERT expression in lung cancer cells, we analyzed the direct interaction of CBP with two transactivators Sp1 and AP-2 bound to hTERT promoter by a co-immunoprecipitation assay. Nuclear extracts from normal lung HLF cells, immotolized HBE cells and lung cancer cells (H1299, A549) were prepared and incubated with a Sp1 or AP-2β antibody. After immunoprecipitation, CBP protein was tested by Western blot. As shown in Fig. 6A, the CBP complexed with Sp1 or AP-2β was detected and considerably increased in lung cancer cell lines compared with that in normal lung cells. These results indicated the specific interaction between CBP and Sp1 or AP-2β in the nucleus of lung cancer cells. Dual immunofluorescence analysis was also used to further analyze the colocalization of CBP with Sp1 and AP-2β(Fig. 6B). The results showed that CBP was colocalized with both Sp1 and AP-2β proteins in H1299 cells in the nucleus. The results indicated again the association between CBP and transactivators Sp1 and AP-2 in lung cancer cells.

### Binding of Sp1 on hTERT promoter in lung cancer cells

It has been shown that the transactivators, such as Sp1 and AP-2, regulate the transcription of hTERT gene [30]. We next selected Sp1 to test its possible binding on the hTERT promoter region through streptavidin-agarose pulldown analysis. The cell nuclear extracts, biotinylated hTERT promoter probes and streptavidin-agarose beads were mixed and incubated. The Sp1 bound on the hTERT promoter probes was detected from the formed complexes by western blot. As shown in Fig. 6C, Sp1 was detected in the complexes from all the four tested cell lines. We also confirmed the binding of Sp1 on hTERT promoter region by ChIP assay using the specific antibody against Sp1. The hTERT promoter was amplified from the Sp1 antibody-immunoprecipitated samples in all the tested cell lines (Fig. 6D). Moreover, we found that the cancer cell lines (A549 and H1299) had the strong binding activity of Sp1 on hTERT promoter by comparison with the normal cell lines (HLF) (Fig. 6C and 6D).

### Acetylation of Sp1 by CBP in lung cancer cells

CBP has a histone acetyltransferase (HAT) activity. To determine whether CBP can acetylate transactivators bound to hTERT promoter, we immunoprecipitated the nuclear extracts from lung normal and cancer cell lines using anti-Sp1 antibody. The acetylation levels of Sp1 was tested using an anti-acetyl-lysine antibody. The comparatively higher level of the acetylated Sp1 protein was detected in lung cancer cell lines (A549 and H1299) than that in normal lung cell lines (HLF) (Fig. 6E).

To determine whether CBP mediated the acetylation of the transactivators bound on hTERT promoter, we tested the effect of CBP on the acetylation level of the transactivator Sp1 in H1299 lung cancer cells. Knockdown of CBP by its specific siRNA or inhibition of CBP activity by its specific inhibitor resulted in a significant decrease in the acetylated level of Sp1 (Fig. 6F). The results indicate that CBP mediates the tumor-specific acetylation of Sp1 in lung cancer cells*.*

We also tested the effect of CBP on the binding of Sp1 to hTERT promoter by pulldown analysis. The comparatively attenuated Sp1 protein was detected in the cells treated with CBP-specific siRNA or inhibitor compared with those in the PBS or non-specific siRNA (NSC-siRNA)-treated cells (Fig. 6G). These results indicate the immortalized and tumor cell-specific binding between hTERT promoter and CBP and Sp1, and potential requirement of CBP in the accumulation of Sp1 at the hTERT promoter.

## DISCUSSION

More and more studies have established the strong positive correlation between the relatively high expression and activation of hTERT and oncogenesis [31-34], and proposed hTERT as a therapeutic target for cancer therapy. Therefore, it is reasonable and significant to reveal the molecular mechanisms of the tumor-specific expression and activation of hTERT. Various cellular factors, including transcription factors and some intracellular signaling molecules, have been confirmed to be able to regulate hTERT expression in cancer cells by binding to hTERT promoter [15-17, 35-37]. The aim of our study was to understand the molecular mechanism of transcriptional regulation for the observed increased expression of hTERT in non-small cell lung cancer cells and further assess its role in non-small cell lung cancer tumorigenesis and development.

With regard to the new insight into the mechanisms involved in hTERT expression regulation in lung cancer, we have demonstrated for the first time that hTERT transcription activation is dependent on CBP. CBP was found to express at significantly higher level in immortalized lung and adenocarcinoma cells than that in normal lung cells *in vitro* and *in vivo* in our study. In addition, our results showed that the level of CBP expression was strongly correlated with the levels of hTERT expression in lung adenocarcinomas specimens, further supporting the importance of CBP in regulation of hTERT expression in lung cancer. Furthermore, the overexpression or knocked down expression of CBP significantly promoted or attenuated hTERT promoter-driven luciferase gene expression in H1299 cell line, partially indicating the positive regulation role of CBP on hTERT promoter. While the streptavidin-agarose pulldown and ChIP assay clearly indicated the direct or indirect binding of CBP to hTERT promoter, the data further approved the regulation of CBP on hTERT promoter.

As a transcriptional co-activator, CBP plays its role to regulate the expression of the targeted genes through mediating the recruitment of basal transcriptional machinery to the promoter either by interacting with these transcriptional factors directly or by acetylating histones and other transcription factors indirectly. Based on the result of the binding of CBP to hTERT promoter from pulldown assay, we hypothesized CBP might regulate hTERT transcription as co-activator in the ways of direct interaction with some special transcriptional factors or acetylating partial transcriptional factors first and then recruiting them to hTERT promoter. The upregulated expression of Sp1 was observed in lung cancer cells than that in normal lung cells in our study (data not shown), and its binding to hTERT promoter was confirmed by pulldown assay in lung adenocarcinomas cells. Furthermore, we also found that the interaction and colocalization between Sp1 and CBP using immunoprecipitation assay and immunofluorescence assay. All of these results suggest that the transcriptional regulation of hTERT expression by CBP might occur through the recruitment of some special transcriptional factors, possibly Sp1 to hTERT promoter region.

In our study, we also found that the inhibition of histone acetyltransferase activity by CBP significantly lowered the binding of Sp1 to hTERT promoter as well as the level of acetylated Sp1, suggesting that the acetylation of Sp1 by CBP might be required to initiate the transcriptional activating mechanism of hTERT promoter in lung cancer cells. More transcriptional factors, besides Sp1, might also be required to lead to transcriptional activation of the hTERT promoter through the recruitment of CBP and binding to hTERT promoter. We will further investigate these questions in our subsequent research.

We also found that CBP was overexpressed in lung cancer cell lines and lung cancer tissues. Importantly, lung cancer patients with strong positive expression of CBP showed shorter survival time after diagnosis when compared to those with weak CBP expression. Even more meaningful, the expression level of CBP was almost positively correlated with hTERT expression level in lung cancer patients, and the knockdown of CBP by siRNA significantly decreased hTERT activity and inhibited lung tumor cell growth, indicating the key role of CBP involved in lung cancer development and progression.

In summary, we found that hTERT induction in lung cancer cells was partially mediated through a mechanism whereby CBP binds and activates the hTERT promoter. We also revealed that CBP interacts with and acetylates Sp1, thereby transactivating hTERT promoter and initiating hTERT expression, which is involved in lung cancer development. Since CBP is nearly not expressed in normal lung cells and highly expressed in lung cancer cells, and its expression level is positively correlated with hTERT expression and inversely proportional to patient survival, it might represent a promising therapeutic target to inhibit hTERT activity and lung tumorigenesis.

## MATERIALS AND METHODS

### Cell lines and cell culture

Human adenocarcinoma cell lines (H1299 and A549), immortalized human lung bronchial epithelial cell line (HBE), and human normal lung fibroblast (HLF), were obtained from the American Type Culture Collection (ATCC, Manassas, VA). H1299 and A549 were cultured in RPMI-1640 medium (HyClone, Thermo Scientific) supplemented with 10% fetal bovine serum. HBE and HLF cells were maintained in Dulbecco's modified Eagle's medium (Invitrogen, Carlsbad, CA) supplemented with 10% fetal bovine serum. All cells were maintained in a humidified atmosphere with 5% CO_2_ at 37°C.

### Western blot analysis

Western blot analysis of the whole cell lysate with an antibody against hTERT (Millipore USA), β-Actin (Cell Signaling Technology, Beverly, MA) and of the nuclear extracts with an antibody against Sp1, AP-2β (Santa Cruz, USA), CBP (Cell Signaling Technology, Beverly, MA) were performed. The protein bands were detected by enhanced chemiluminescence (Amersham Pharmacia Biotech, Piscataway, NJ) according to the manufacturer's instructions.

### Plasmid vectors

Recombinant plasmid vectors pGL3-hTERT-438 expressing luciferase driven by a hTERT promoter (-378 to +60) were constructed in our lab and used in the transfection experiments. The CBP overexpression vector, pcDNA3.1-CBP or control vector pcDNA3.1-Lac Z plasmids were designed and synthesized by Cyagen (Cyagen Biosciences Inc., United States).

### Transient transfection of lung cancer cells

To overexpress CBP in H1299 cells, pcDNA3.1-CBP or control vector plasmids were transfected with Lipofectamine 2000 (Invitrogen, Carlsbad, CA). To inhibit CBP expression, H1299 cells were transfected with CBP specific siRNA (10μmol/L, Santa Cruz, sc-29244, siRNA-1 and OriGene, SR300976, siRNA-2); nonspecific siRNA (10μmol/L, Santa Cruz, sc-44230). Forty-eight hours post-transfection, cytoplasm and nuclear fractions were isolated [28], and proteins were tested using western blot analysis.

### Treatment of lung cancer cells with CBP inhibitor

To inhibit the histone acetyltransferase (HAT) activity of CBP in H1299 cells, c646, a competitive HAT CBP inhibitor (Sigma-Aldrich, SML0002), was used to treat H1299 cells. 48 hours after treatment, cytoplasm and nuclear fractions were isolated, and protein activities were analyzed.

### Promoter activity and dual-luciferase assay

Cells (2×10^5^ cells/well) were seeded into six-well plates, cultured overnight, and transfected with the hTERT promoter-luciferase plasmids (1μg pGL3-hTERT-400 plasmids per well) mediated by Lipofectamine 2000 (Invitrogen, Carlsbad, CA). Meanwhile, cells were cotransfected with either CBP overexpression vector or CBP specific siRNA (pcDNA3.1-CBP, or control vector pcDNA3.1-Lac Z plasmids; CBP specific siRNA or nonspecific siRNA). Transfection efficiency was normalized by co-transfection with Renilla luciferase reporter. Both firefly and Renilla luciferase activity were quantified using a dual-luciferase assay system (Promega Corp., Madison, WI).

### Telomerase activity assays

Telomerase activity was analyzed by telomerase PCR enzyme-linked immunosorbent assay kit (Roche Applied Science) as described previously [28].

### Immunofluorescence and confocal microscopy

Cells were fixed with 4% paraformaldehyde (w/v) for 4 min, quenched for 20 min with 50 mM NH_4_Cl in PBS and permeabilized with 0.2% (w/v) TritonX-100 in PBS for 5min. The blocking step was performed for 30 mins in PBS containing 10% bovine serum albumin (BSA) (w/v). Cells were then incubated overnight with the primary antibodies against CBP, Sp1 diluted in PBS containing 1% BSA. After PBS washings, cells were incubated for 2 h with secondary antibodies conjugated with Alexa Fluor 488 or Alexa Fluor 555. CBP and Sp1 protein localization was assessed using a Leica confocal microscopy (Model TCS-NT). Files of microphotographs were processed with the Adobe Photoshop 5.0 software (San Jose, CA, USA).

### Co-immunoprecipitation assays

Equal amounts of nuclei protein extracts prepared from different cell lines were incubated with the antibodies against Sp1, AP-2β and CBP for 24 h at 4°C. Then, the agarose-conjugated protein-A/G beads (Santa Cruz Biotech) were added and the mixture was incubated at 4°C for another 12h. After extensive washing with cold phosphate-buffered saline (PBS), the beads were mixed with loading buffer and boiled. The proteins in the supernatant were separated by SDS-PAGE and transferred to PVDF membranes for Western blotting analysis.

### Streptavidin-agarose pulldown assay

Binding of transcriptional factors or co-activators to hTERT promoter DNA was assayed by streptavidin-agarose pulldown using a biotin-labeled double-strand DNA probes corresponding to hTERT promoter sequence −378 to +60 and synthesized by Sigma-aldrich (St.Louis, MO) as described previously [28]. Briefly, cells were grown to 80-90% confluence in 150-cm^2^ flasks and nuclear extracts were prepared [29]. The binding assay was performed by mixing 400μg nuclear extract proteins, 10ug DNA probes, and 100μl streptavidin-agarose beads (Sigma-aldrich, American). The mixture was incubated at room temperature for 6h with shaking and centrifuged at 500g to pull down the DNA-protein complex. The bound proteins were washed by cold PBS three times and further eluted by being boiled at 95°C for 5 mins for Western blot analysis.

### Chromatin immunoprecipitation assay (ChIP)

The ChIP assay was performed using the ChIP IT Express kit (Active Motif, Rixensart, Belgium) following the manufacturer's instructions. Briefly, the cells were “fixed” with 1% formaldehyde and sonicated on ice to shear the DNA to 200 bp to 500 bp. One-third of the total cell lysate was used as the DNA input control. The remaining two thirds of the lysate were subjected to immunoprecipitations with anti-CBP, anti-Sp1 antibodies or non-immune rabbit IgG. The DNA was subjected to PCR to amplify a 438 bp region (-378 to +60 bp) of the hTERT promoter using the primers (5′- TGGCCCCTCCCTCGGGTTAC-3′ and 5′- CCAGGGCTTCCCACGTGCGC-3′). The PCR products were resolved electrophoretically on a 2% agarose gel and visualized by ethidium bromide staining.

### Cell viability assay

Cell viability was determined by MTT assay (Roche Diagnosis, Indianapolis, IN) according to the manufacturer's protocol. Briefly, H1299 cells plated in 96-well plates (2000 cells/well) were treated with pcDNA3.1-CBP, or control vector pcDNA3.1-Lac Z, CBP siRNA or control siRNA (50 nmol/L). At different time points after treatment, cell viability was determined.

### *In vivo* tumor model and tissue processing

Animal experiments were carried out in accordance with the National Institute of Health Guide for the Care and Use of Laboratory Animals, with the approval of the Animal Research Committee of Dalian Medical University. CBP siRNA and negative control siRNA for *in vivo* delivery were obtained from Santa Cruz Co (USA). To investigate the effect of CBP inhibition on lung cancer cell growth *in vivo*, A549 cells (2×10^6^) were inoculated subcutaneously into the flank of the nude mice. Mice were randomly divided into 2 groups (5 mice per group): (a) control siRNA and (b) CBP siRNA. Treatment was started 2 weeks after injection. For delivery of siRNA complexed with liposome, the complexes with 10 nmol RNA in 0.1 ml 5% glucose were injected intratumorally twice a week for 3 weeks. The tumor volume in mm^3^ was calculated as V = (width^2^ × length)/2 using digital calipers and the tumor weight was recorded after the mice were sacrificed. Tumor specimens were fixed in formalin and embedded in paraffin for CBP and hTERT protein expression analysis through the immunohistochemical staining described below.

### Human lung adenocarcinoma specimens

Human lung adenocarcinomas tissue microarray was purchased from Shanghai Outdo Biotech (Shanghai, China) and contains 150 lung adenocarcinomas and their corresponding adjacent non-malignant normal lung tissues. The immunostaining analysis of CBP and hTERT protein expression was done based on these tissue microarrays. The extent of the staining was used as criteria of evaluation. For each tissue sample, protein expression was scored according to the staining color: negative staining (no yellow); low staining(light yellow); moderate or high staining (yellowish brown or brown). Overall survival (OS) was calculated from the day of surgery to the day of death or to the last follow-up day. With prior written consent from patients, all the tissue samples had been obtained before anticancer treatment.

### Immunohistochemistry staining

The tissue microarray (TMA) slides were deparaffinized in xylene and rehydrated through graded alcohol. Antigen retrieval was performed by incubating samples with citrate buffer (0.1mol/L, pH 6.0) for 90 minutes (for hTERT detection) and with Target Retrieval Solution (pH 9; DakoCytomation) for 15 minutes (for CBP detection) using a pressure cooker. The slides were then immersed in methanol containing 3% hydrogen peroxide for 20 minutes to block endogenous peroxidase activity. After preincubation in 2.5% blocking serum to reduce nonspecific binding, the sections were incubated overnight using a primary antibody, either anti-CBP (#7389, CST, 1:50 dilution), or anti-hTERT antibody (#MABE14, MILLIPORE, 1:50 dilution), in a humidified container at 4°C. The TMA slides were processed with horseradish peroxidase immunochemistry according to the manufacturer's recommendations (DakoCytomation, Carpinteria, CA). As a negative control, the staining procedure was performed with the primary antibody replaced by a normal rabbit IgG. Since CBP and hTERT protein were mainly detected in the nuclei of cancer cells, nuclear staining intensity was graded as: absent staining as 0, weak as 1, moderate as 2, and strong as 3. The percentage of stained cells was graded as: 0 (no positive cells), 1 (<25% positive cells), 2 (25%–50% positive cells), 3 (50%–75% positive cells), and 4 (>75% positive cells). The score for each tissue was calculated by multiplying the intensity and the percentage value (range, 0–12). The median immunohistochemical staining score was used as the cutoff value for tumor “high expression”. CBP and hTERT expression were classified as high level (score ≥ 7) or low level (score ≤ 6).

### Statistical analysis

Student's t-test was used to compare two independent groups of data. Chi-square tests were applied to analyze the association between CBP and hTERT abundance. Survival curves were constructed using the Kaplan-Meier method and were compared using the log-rank test. Statistical analyses were performed using SPSS 16.0 software. Results were shown as mean ± SE. *P* < 0.05 was considered to be significant.
